# Prevailing Diseases

**Published:** 1883-02-20

**Authors:** 


PREVAILING DISEASES.—The extraordinary changes of temperature alterations, with dampness and rain, have tended to the production of bronchial and throat affections during the last two or three months. Occasional cases of pneumonia with neuralgias and typho-malarial fevers have prevailed in many localities.

